# Chemical Composition and Biological Activity of Extracts from *Salvia bicolor* Desf. Growing in Egypt

**DOI:** 10.3390/molecules171011315

**Published:** 2012-09-25

**Authors:** Taghreed A. Ibrahim

**Affiliations:** Pharmacognosy Department, College of Pharmacy, King Saud University, P.O. Box 22452, Riyadh 11495, Saudi Arabia; Email: tshehata@ksu.edu; Tel.: +966-55-663-9972; Fax: +966-1-206-2138

**Keywords:** phenolic acids, flavonoids, antioxidant, antimicrobial, anti-inflammatory, analgesic

## Abstract

A petroleum ether extract (PEE) and a methanolic extract (ME) of aerial parts of *Salvia bicolor* Desf were prepared, and their chemical compositions and antioxidant, anti-inflammatory, analgesic and antimicrobial activities were evaluated. GC/MS analysis of the PEE revealed the presence of 20 compounds in the unsaponifiable matter, among which β-sitosterol and β-amyrin (constituting 24.75% and 15.62%, respectively) were the main constituents, and 21 fatty acids, with linolenic acid and erucic acid being the main fatty acid constituents (21.65% and 16.65%, respectively). HPLC/MS analysis of the methanol extract for individual phenolics revealed the presence of 14 phenolic acids; protocatchuic acid was predominant (75.22 mg/g dry sample), followed by *p*-coumaric, gallic and synergic acids (70.27, 68.26 and 54.38 mg/g dry weight, respectively). HPLC/MS analysis of flavonoid contents revealed the presence of five flavonoid compounds, among which luteolin 7-*O*-glucoside and apigenin were the major constituents (120.25 mg/100g dry sample and 88.48 mg/100g dry sample, respectively). The antioxidant activities of both extracts were evaluated using the 2,2-diphenyl-1-picrylhydrazyl (DPPH) assay, and the total antioxidant capacity was determined in terms of GAE (gallic acid equivalents). Anti-inflammatory and analgesic activities were evaluated using the rat paw edema and hot plate testing methods, respectively. The antimicrobial activities of both the PEE and ME were examined by means of the disk-diffusion method. The *Salvia bicolor* PEE and ME exhibited significant antioxidant, anti-inflammatory, and analgesic properties, in addition to antimicrobial effects against the selected microorganisms.

## 1. Introduction

The genus *Salvia* (commonly known as sage) is a broad genus belonging to the family Lamiaceae*,* which is a large cosmopolitan family of approximately 252 genera and 7,200 species [1,2]. Several species of *Salvia* are cultivated for their aromatic characteristics and are used as flavorings, food condiments, cosmetics and perfume additives [3]. Additionally, *Salvia* species have commonly been widely used as folk medicines as antibacterial, antiviral, antitumor, spasmolytic, antioxidant and anti-inflammatory treatments and have further been used in the treatment of mental, nervous and gastrointestinal conditions [4,5].

Phytochemical studies conducted on plants of this genus have led to the isolation of numerous diterpenoids of the abietane, ictexane, labdane, neoclerodane and phenalenone types [6,7,8]. Triterpenes and sterols were also reported [9], in addition to anthocyanins, coumarins, polysaccharides, flavonoids and phenolic acids and their derivatives [4]. Several studies have investigated the antioxidant activities of *Salvia* species [5,10,11,12,13], including screening of the antioxidant potential of some *Salvia* extracts and essential oils. Moreover, the anti-inflammatory [13,14], analgesic and antipyretic [15], antiepileptic, anti-ulcerogenic and tranquillizing activities [16] and antimicrobial effects [5,17,18,19,20,21] of different *Salvia* species have also been studied.

Traditionally, medicinal plants are used throughout the world for a range of pain complications. Plant drugs are frequently considered to be less toxic and free of side effects than synthetic ones. The study of such medicines might offer a natural key to alleviating of pain for the future. *Salvia* is an important genus widely cultivated and used in flavoring and folk medicines. *Salvia* species are used as traditional medicines all around the world, possessing antibacterial, antioxidant, anti-inflammatory and analgesic properties. 

In Egypt, the genus *Salvia* is represented by ten species [22]. *Salvia bicolor* Desf., a species mainly indigenous to Mexico and North Africa, is a biennial herb and flowers from May to July, bearing long close spikes with pale violet blue flowers [23]. As far as could be ascertained via a literature survey, investigations of the biological properties and chemical composition of *S. bicolor* have not been previously reported. Thus, this study could be assumed to represent the first report of a biological and chemical investigation of *S*. *bicolor* Desf. The aim of this study was to identify the chemical composition of petroleum ether and methanol extracts from aerial parts of *S. bicolor* and to evaluate their antioxidant, anti-inflammatory, analgesic and antimicrobial activities.

## 2. Results and Discussion

### 2.1. Composition of the Unsaponifiable Fraction and Fatty Acids from a Petroleum Ether Extract

GC/MS analysis of the unsaponifiable fraction revealed the presence of 20 compounds (Table 1). This fraction was characterized by large amounts of hydrocarbons, which constituted 48.05% of the fraction, and octacosane, with a MW of 394 and a base peak of *m/z* 57, was the major one (11.01%). Two oxygenated hydrocarbons were detected: isophytol (2.15%) and phytol (1.86%). Sterols represented 27.85% of the total unsaponifiable fraction; the major sterol was β-sitosterol (24.75%), with the molecular formula C_29_H_50_O, a MW of 414 and a base peak of *m/z* 43. Two triterpenoids were identified by GC/MS analysis of the unsaponifiable fraction: β-amyrin (15.62%), C_30_H_50_O, with a MW of 426 and base peak of *m/z* 207, and lupeol (4.47%), C_30_H_50_O, with a MW of 426 and a base peak of *m/z* 43. Identity was confirmed by comparison with published data [24].

**Table 1:** GC/MS analysis of unsaponifiable matter in the *S*.* bicolor* PEE.

Peak	R_t_ (min)	%	M^+^	Base peak	Compound name
1	18.99	0.50	156	57	Undecane
2	20.12	0.30	170	57	Dodecane
3	23.83	4.31	226	43	Hexadecane
4	26.7	1.40	240	57	Heptadecane
5	27.32	2.10	268	57	Nonadecane
6	28.9	2.13	282	57	Eicosane
7	30.7	7.70	296	57	Heneicosane
8	31.29	2.15	296	71	Isophytol
9	31.74	1.86	296	71	Phytol
10	32.10	2.50	310	57	Docosane
11	33.70	2.10	324	57	Tricosane
12	35.04	0.70	410	69	Squalene
13	36.20	10.10	366	57	Hexacosane
14	37.27	3.20	380	57	Heptacosane
15	38.80	11.01	394	57	Octacosane
16	39.80	0.70	400	43	Campesterol
17	40.30	2.40	412	55	Stigmasterol
18	41.04	24.75	414	43	β-Sitosterol
19	41.5	4.47	426	43	Lupeol
20	43.64	15.62	426	207	β-amyrin

These results are in agreement those reported for several *Salvia* species in which pentacyclic triterpenoids are common [25]. Both α- and β-amyrins were found in *S*. *amplexicaulis* and *S. apiana* [26], β-amyrin, lupeol, β-sitosterol, stigmasterol were also detected in *S*. *aegyptiaca* [27].

GC/MS analysis of fatty acids as methyl esters enabled the identification of 21 fatty acids (Table 2). Unsaturated fatty acids represented 41.86% of the total fatty acid fraction of the PEE, where linolenic acid (21.65%) and erucic acid (16.65%) were the major fatty acids detected. The high percentage of unsaturated fatty acids in PEE supports its high antioxidant activity. Linoleic, linolenic and oleic acids were found as major fatty acids in several *Salvia* species [28], while the high percentage of erucic acid in this study is characteristic for *S. bicolor*.

**Table 2:** GC/MS analysis of the fatty acid methyl esters in the *S*. *bicolor* PEE.

Peak	R_t_ (min)	%	M^+^	Base peak	Compound name
1	3.24	1.7	130	74	Methyl ester of caproic acid (C6:0)
2	3.67	0.37	158	74	Methyl ester of caprylic acid (C8:0)
3	5.46	1.86	172	74	Methyl ester of pelargonic acid (C9:0)
4	5.87	1.22	200	74	Methyl ester of undecylic acid (C11:0)
5	6.30	0.94	214	74	Methyl ester of lauric acid (C12:0)
6	7.22	2.13	228	74	Methyl ester of tridecylic acid (C13:0)
7	8.56	7.8	242	74	Methyl ester of myristic acid (C14:0)
8	9.83	1.65	256	74	Methyl ester of pentadecylic acid (C15:0)
9	10.31	10.6	270	74	Methyl ester of palmitic acid (C16:0)
10	11.07	2.8	284	74	Methyl ester of margaric acid (C17:0)
11	11.68	1.85	298	74	Methyl ester of stearic acid (C18:0)
12	12.50	1.63	296	55	Methyl ester of oleic acid (C18:1)
13	14.88	0.38	294	67	Methyl ester of linoleic (C18:2)
14	16.28	21.65	292	79	Methyl ester of linolenic acid (C18:3)
15	18.2	12.85	326	74	Methyl ester of arachidic acid (C20:0)
16	20.58	0.43	340	74	Methyl ester of heneicosylic acid (C21:0)
17	22.67	2.7	354	74	Methyl ester of behenic acid (C22:0)
18	24.32	16.65	352	55	Methyl ester of erucic acid (C22:1)
19	26.11	1.55	350	67	Methyl ester of docosadienoic acid (C22:2)
20	27.56	6.33	368	74	Methyl ester of tricosylic acid (C23:0)
21	28.53	2.91	382	74	Methyl ester of lignoceric acid (C24:0)

### 2.2. Determination of the Total Phenol Content

The total phenol content was determined using the Folin-Ciocalteu reagent [29] in comparison with standard gallic acid, and the result was expressed in terms of mg GAE/g of dry sample. The total phenol content value for the ME from the aerial part of *S*. *bicolor* was 326.76 mg ± 1.62 GAE/g dry sample. The study proved that *S. bicolor* contains a considerably high content of total phenols comparing to other *Salvia* species. As far as our literature survey could ascertain, several studies have been carried out with the *Salvia* species and total phenolic content has been found to be between 41–176.1 mg GAE/g dry weight basis [30,31,32].

### 2.3. HPLC Analysis of Phenolic Acids

HPLC analysis of the phenolic acids in the methanol extract from the aerial part of *S*. *bicolor* (Table 3) showed the presence of 14 phenolic acids. Protocatchuic acid was the predominant phenolic compound (75.22 mg/g dry sample), followed by *p*-coumaric acid, gallic acid and syringic acid (70.27, 68.26 and 54.38 mg/g dry sample, respectively). The major phenolic acids identified were previously detected in other *Salvia* species [33]. Protocatchuic acid was isolated from *S. miltiorrhiza* [34], and together with rosmarinic acid and caffeic acid was proved to possess antioxidant and antimicrobial activities [35]. Gallic, caffeic, chlorogenic and rosemarinic acids were the major phenolic acids detected in *S. verticellata*, *S. trichoclada* and *S. korenenburgii* [36] and *p*-coumaric, caffeic, ferulic, and sinapic acids were found to occur in *Salvia splendens* [37].

**Table 3:** Phenolic acid content of the methanolic extract from the aerial parts of *S. bicolor*.

Peak	R_t_ (min)	Concentration (mg/g dry sample)	Compound name
1	2.3	68.26 ± 1.03	Gallic acid
2	2.8	25.44 ± 0.78	Sinapic acid
3	4.8	3.48 ± 0.83	Caffeic acid
4	6.3	0.72 ± 1.02	Ferulic acid
5	8.1	0.42 ± 1.65	*o*-Coumaric acid
6	9.3	0.78 ± 0.45	*trans*-Cinnamic acid
7	10.8	70.27 ± 0.74	*p*-Coumaric acid
8	11.9	75.22 ± 1.41	Protocatchuic acid
9	12.2	1.44 ± 1.85	*m*-Coumaric acid
10	13.5	0.53 ± 1.06	Chlorogenic acid
11	15.7	0.50 ± 0.92	Gentisic acid
12	20.9	54.38 ± 1.26	Syringic acid
13	21.5	0.68 ± 1.73	*p*-Hydroxybenzoic acid
14	22.0	0.45 ± 0.65	Salicylic acid

Notes: Values are the mean ± SD (n = 3).

### 2.4. Determination of Flavonoid Contents

Colorimetric estimation of the total flavonoids in the ME calculated based on quercetin was 0.45 g/100 g dry sample. HPLC analysis of the ME (Table 4) revealed the presence of five flavonoid compounds. Atanassova *et al.* [38] have studied the flavonoid content in *S*. *officinalis*, which was calculated to be 27.54 mg/100 g dry weight, while Szentmihalyi and Then [39] have reported that flavonoid content in different *Salvia* species were extremely variable, ranging from 2.91% in Hungarian *S*. *officinalis* to 7.85% in Hungarian *S*. *nemorosa*. The great variation in flavonoid content in different *Salvia* species may be due to hereditary factors, habitat, season of collection and different techniques used in evaluation.

**Table 4:** Flavonoid content of the methanolic extract from aerial parts of *S. bicolor*.

Peak	R_t_ (min)	Concentration (mg/100 g dry sample)	Mass Fragments (m/z)	Compound name
1	23.2	120.25 ± 0.35	287, 285	Luteolin-7- *O*-glucoside
2	40.8	54.96 ± 1.18	328, 313, 299, 285, 282, 153	Salvigenin
3	47.3	52.34 ± 0.97	273, 257, 179, 151	Quercetin
4	64.2	42.30 ± 0.56	267, 243, 241, 217, 151, 107	Luteolin
5	87.4	88.48 ± 1.32	269	Apigenin

Notes: Values are the mean ± SD (n = 3).

Analysis of the mass spectra of the five compounds and comparison of their chromatographic behavior with that of authentic samples, in addition to comparison with the available literature [40,41,42,43,44] allowed them to be characterized (Table 4). Luteolin 7-*O*-glucoside (120.25 mg/100 g dry sample) was the only glycoside detected and constitutes the major flavonoid present; this compound was previously identified in other *Salvia* species [45]. Salvigenin, which exhibited a concentration of 54.96 mg/100 g dry sample, is a trimethoxylated flavone aglycone that was previously isolated from numerous *Salvia* species [46,47]. Additionally, quercetin (52.34 mg/100 g dry sample), a flavonol aglycone, and two flavone aglycones, luteolin (42.30 mg/100 g dry sample) and apigenin (88.48 mg/100 g dry sample), were detected, which have previously been characterized in many *Salvia* species [48,49,50]. These five flavonoid compounds, which were previously isolated and identified in numerous *Salvia* species, were detected for the first time in *S. bicolor*. Luteolin 7-*O*-glucoside, the major flavonoid detected in *S. bicolor* was proved to have significant antimicrobial [51], antiasthmatic [52], anti-inflammatory and antinociceptive [53] activities, in addition to a potent antioxidant effect [54].

### 2.5. DPPH Radical-Scavenging Activity

**Figure 1:**
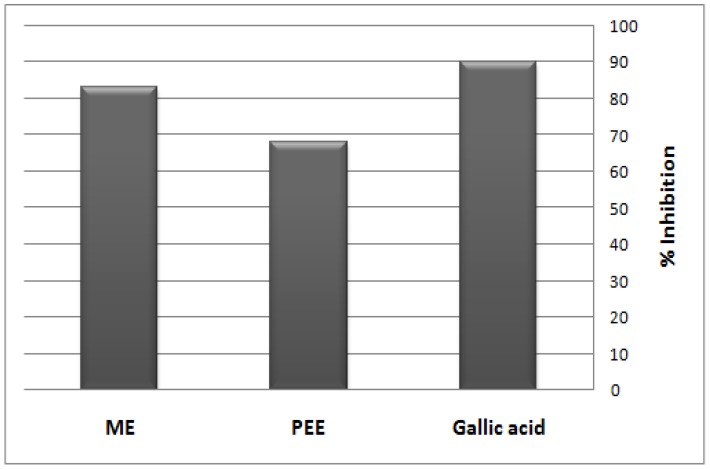
DPPH radical-scavenging activity of the PEE and ME from aerial parts of *S. bicolor* relative to standard gallic acid.

DPPH is a free radical compound that has widely been used to test the free radical scavenging abilities of various types of samples [55,56]. It is a stable free radical showing a characteristic absorption at 517 nm. Upon interaction with DPPH, antioxidants transfer either an electron or a hydrogen atom to DPPH, thus neutralizing its free radical character [57] and the color of the compound changes from purple to yellow, and its absorbance at a wavelength of 517 nm decreases. To evaluate the DPPH-scavenging effects of the PEE and ME from *S. bicolor* aerial parts, DPPH inhibition was investigated. The results are shown as the relative activities against standard gallic acid (Figure 1). The activity of the ME (2500 µg/mL) was higher than that of the PEE (2,500 µg/mL) and was nearly similar to that of 250 µg/mL of gallic acid.

The plant extracts under investigation quenched DPPH in a dose-dependent manner (r^2^ = 0.893 and 0.964 for the PEE and ME, respectively). Similar results were also found in terms of IC_50_ values; the IC_50_ values (concentration of the sample required to scavenge 50% of the free radicals) were found to be 393.00 µg/mL and 321.28 µg/mL for the PEE and ME, respectively, which demonstrated the higher antioxidant activity of the ME over the PEE. The high DPPH free radical scavenging effect of ME may be attributed to its content of flavonoids and phenolic acids, as protocatchuic, ferulic and caffeic acids were proved to have antioxidant activity [40,58] while the relatively high effect of PEE may be due to its high content of unsaturated fatty acids (41.86%) [59].

### 2.6. Determination of the Total Antioxidant Capacity

The total antioxidant capacity of the PEE and ME from *S. bicolor* aerial parts was expressed as the number of equivalents of gallic acid. This assay is based on the reduction of Mo (VI) to Mo (V) by the extract and the subsequent formation of a green phosphate/Mo (V) complex at an acidic pH. The phosphomolybdenum method is quantitative, as the antioxidant activity is expressed as the number of equivalents of gallic acid [60,61]. The ME (350 mg GAE/g dry sample) showed higher antioxidant activity than the PEE (130 mg GAE/g dry sample). The ME showed higher antioxidant activity than the PEE. The effect of the ME might be due to its content of phenolic acids and flavonoids, which have been demonstrated to exhibit antioxidant activity [40,58], whereas the antioxidant activity of the PEE could be attributed to its high content of unsaturated fatty acids [59].

### 2.7. Determination of Anti-inflammatory Effects

The results of experiments examining the effects of the PEE and ME from *S. bicolor* aerial parts on carrageenan-induced paw edema are presented in Table 5.

**Table 5:** Anti-inflammatory effect of the PEE and ME from *S*. *bicolor* aerial parts on carrageenan-induced rat paw edema.

Group	After 1 h		After 2 h		After 3 h		After 4 h	
	Edema (mm)	% inhibition	Edema (mm)	% inhibition	Edema (mm)	% inhibition	Edema (mm)	% inhibition
Control (saline)	78.2 ± 0.5	--	95 ± 0.6	----	110 ± 0.6	---	113 ± 0.7	---
PEE ^a^	70.5 ± 1.3	9.8 ± 1.5	77.8 ± 2.1	18.1 ± 1.6	84.5 ± 1.7	23.2 ± 2.9	80.3 ± 1.7	28.9 ± 1.8
ME ^a^	60.2 ± 0.20	23 ± 0.22	77.8 ± 2.0	18.1 ± 2.07	77.65 ± 0.7	29.4 ± 2.7	73 ± 0.6	35.4 ± 1.4
Indomethacin^b^	70.1 ± 1.5	10.4 ± 1.3	77.0 ± 0.5	18.9 ± 0.20	75 ± 1.6	31.2 ± 1.7	75 ± 0.6	33.6 ± 1.2

Data are presented as the mean of three experiments ± SD; % inhibition was calculated as significant changes from control values at each respective time. ^a^ Dose: 50 mg/kg b. wt. ^b^ Dose: 10 mg/kg b. wt.

These assays revealed that oral administration of 50 mg/kg b. wt. PEE and ME reduced paw edema by 23.2% and 29.4% after three hours, respectively. The percentages of inhibition were 28.9% and 35.4% after 4 h for the PEE and ME, respectively. The results showed that the ME exerted a stronger effect than the standard anti-inflammatory drug indomethacin after both 1 h and 4 h of administration. These results were in agreement with findings previously reported for several *Salvia* species [13,14,16]. This activity might be due to the presence of flavonoids [62] and phenolic compounds, which have been reported to possess anti-inflammatory activities, especially gallic, *p*-coumaric and protocatchuic acids, which are present in high percentages in the *S. bicolor* ME [63,64,65,66]. The significant anti-inflammatory effect of the PEE from *S. bicolor* aerial parts might be attributed to the high percentages of β-sitosterol (24.75%) which has proved to possess a potent anti-inflammatory activity [67], as well as the presence of β-amyrin (15.62%) which has showed a significant anti-inflammatory [68], in addition to the presence of lupeol (4.47%) which has proved to have anti-inflammatory, analgesic and antipyretic effects [69].

### 2.8. Determination of Analgesic Effects

Oral administration of the PEE and ME from *S. bicolor* aerial parts significantly increased hot plate latency, indicating the analgesic effect of both the PEE and ME (Table 6). The PEE administered at a dose of 50 mg/kg b.wt. increased hot plate latency at 1 h and 2 h post-treatment compared with the pre-drug basal value (time 0). Treatment with the ME at a dose of 50 mg/kg b.wt. also increased hot plate latency at 1 h and 2 h post-treatment compared with the pre-drug basal value (time 0). Several *Salvia* species have been demonstrated to possess analgesic activities [70]. The analgesic effects of the ME of *S. bicolor* might be attributed to the presence of flavonoids and phenolic acids [71,72,73]. Additionally, the PEE analgesic effect may be due to the presence of high percentages of β-amyrin [74], lupeol [69] and β-sitosterol [75].

**Table 6:** Analgesic effect of the PEE and ME from *S. bicolor* aerial parts determined via a hot plate test.

Group	Pre-drug treatment	After 1 h		After 2 h	
	M ± SD	M ± SD	% of change	M ± SD	% change
Control saline	20 ± 0.97	21.85 ± 0.21	--	20.85 ± 2.1	---
PEE ^a^	20.68 ± 0.47	27.85 ± 0.86	34.7 ± 1.3	32.6 ± 2.1	57.6 ± 1.6
ME ^a^	19.33 ± 2.4	30.85 ± 0.49	59.6 ± 1.67	32.48 ± 2.2	68 ± 1.5
Indomethacin ^b^	18.33 ± 0.69	23.5 ± 1.66	28.2 ± 1.66	28.9 ± 1.4	57.7 ± 1.2

M ± SD: mean of three experiments ± standard deviation; % change: calculated as significant changes from pre-drug treatment values. ^a^ Dose: 50 mg/kg b. wt. ^b^ Dose: 10 mg/kg b. wt.

### 2.9. Determination of Antimicrobial Activity

The antimicrobial activities of the PEE and ME from the aerial parts of *S. bicolor* are shown in Table 7. The PEE showed antibacterial activities against G +ve bacteria, with the strongest effect being detected against *Staphylococcus epidermidis*. The PEE exhibited a moderate effect against G –ve, bacteria, whereas it showed strong effect against *Candida albicans*. The ME showed strong antimicrobial activities against G +ve bacteria, especially *Staphylococcus aureus* and *Staphylococcus epidermidis*, but a moderate effect on G –ve bacteria, and it possessed high antimicrobial activity against *Candida albicans*. The antimicrobial activities of the PEE and ME might be attributed to their contents of terpenoids [76] or flavonoids and phenolic acids [77,78], respectively.

The results of this study are in agreement with observations made in other studies. For example, methanolic extracts from *S. cryptantha* and *S. multicaulis* also exhibited antimicrobial potential [5]. An acetone extract from *S. jaminiana*, containing the sterols campesterol, stigmasterol andsitosterol as well as five diterpenoids was found to markedly inhibit the growth of G +ve bacteria [18]. An antimicrobial analysis of *S. chamelaeagnea* extracts showed strong antibacterial activity against both G +ve and G –ve bacteria [19]. It was found that the dichloromethane fractions from acetone extracts of *S. sclarea* roots as well as four pure diterpenes isolated from hairy root cultures presented antimicrobial activity against G +ve bacteria. Additionally, a methanolic extract from *S. pisidica* [20] showed high antimicrobial activity against G +ve bacteria and a moderate effect against G –ve species, and an ether extract from *S. lanigera* growing in Egypt, which contained several flavonoid compounds, exhibited marked antimicrobial activity against G +ve bacteria, but a weaker effect on G –ve microorganisms [21].

**Table 7:** Antimicrobial activity and MIC of the PEE and ME from *S. bicolor* determined using agar disc diffusion and MIC methods.

Microorganism	PEE		ME		MICs of the standards	
	DD ^a^ M ± S.D.	MIC ^b^	DD ^a^ M ± S.D.	MIC ^b^	Gentamycin	Amphotericin B
*Staphylococcus aureus*	13.2 ± 0.44	550	18.3 ± 0.92	200	8 × 10^−3^	NT
*Staphylococcus epidermidis*	14.6 ± 1.17	650	16.8 ± 1.27	350	1 × 10^−2^	NT
*Streptococcus pyogens*	10.7 ± 0.56	1,000	14.3 ± 0.64	350	8 × 10^−3^	NT
*Escherichia coli*	9.1 ± 0.24	1,000	12.7 ± 1.84	400	8 × 10^−3^	NT
*Klebsiellapneumonia*	7.2 ± 1.76	1,000	9.58 ± 1.26	1000	1 × 10^−2^	NT
*Proteusvulgaris*	8.8 ± 0.77	1,000	9.3 ± 0.60	1000	1 × 10^−2^	NT
*Pseudomonas aeruginosa*	11.4 ± 0.54	900	11.4 ± 0.70	400	1 × 10^−2^	NT
*Shigellaboydii*	10.2 ± 1.28	1,000	13.6 ± 0.62	400	1 × 10^−2^	NT
*Candida albicans*	13.2 ± 0.43	400	16.4 ± 1.30	350	NT	1 × 10^−3^
*Candida glabrata*	7.2 ± 0.76	1,000	7.3 ± 1.40	1000	NT	1 × 10^−3^
*Candida krusei*	8.2 ± 0.93	1,000	8.5 ± 1.87	1000	NT	1 × 10^−3^
*Candida parapsilosis*	8.5 ± 0.36	1,000	8.5 ± 0.85	1000	NT	1 × 10^−3^

^a^ DD, agar disc diffusion method. Diameter of the inhibition zone (mm) including the disc diameter of 6 mm; ^b^ MIC, minimum inhibitory concentration; values are given as µg/mL; NT, not tested; M ± S.D., mean of three experiments ± standard deviation.

The results showed that methanol extract of *S. bicolor* is more active than petroleum ether extract as antioxidant, anti-inflammatory and analgesic and antimicrobial. The results obtained in this study are in agreement with previous investigations on *Salvia* species. For example, the antimicrobial and antioxidant effects of methanol extract of S. tomentosa was superior to non-polar extracts [17]. It was found that methanol extract of *S. officinalis* is more active than petroleum ether extract as anti-inflammatory [79]. Additionally, the methanol extract of *S. hypolecuca* was found more effective as antioxidant than that of petroleum ether extract [80].

## 3. Experimental

### 3.1. Plant Material

Aerial parts of *Salvia bicolor* were collected from Saint Catherine in South Sinai, Egypt, during the flowering stage in May 2009 and kindly identified by Dr. Moneer Abd El-Ghany, Professor of Plant Taxonomy, Faculty of Science, Cairo University. A voucher specimen has been deposited in the Pharmacognosy Department, Faculty of Pharmacy, Cairo University, Egypt. The plant material was air dried to constant weight, powdered and kept in tightly closed amber-colored glass containers, protected from light at as low a temperature as possible.

### 3.2. Preparation of the Petroleum Ether Extract (PEE)

Air dried aerial parts of *S. bicolor* (250 g) were exhaustively extracted with petroleum ether (1 L) at 60–80 °C for 24 h in a continuous extraction apparatus, and the extract was evaporated under vacuum to yield 0.5 g% of dry extract.

### 3.3. Analysis of the Petroleum Ether Extract

#### 3.3.1. Preparation of Unsaponifiable Matter [81,82]

One gram of the PEE was saponified via reflux with 10% alcoholic KOH (100 mL) for 5 h. The liquid was distilled off almost to dryness. The residue was suspended in water (100 mL) and extracted with chloroform until achieving complete extraction of the unsaponifiable matter. The combined chloroformic extract was washed with distilled water to remove any alkalinity present, dried over anhydrous sodium sulphate and evaporated to dryness (0.52 g).

#### 3.3.2. Preparation of Fatty Acid Methyl Esters

The alkaline aqueous layer remaining after the extraction of unsaponifiable matter was acidified with dilute hydrochloric acid. The liberated fatty acids were extracted with successive volumes of ether. The combined ethereal extract was washed with distilled water, dried over anhydrous sodium sulphate, evaporated to dryness (0.37 g) and methylated via reflux for 90 min with methanol-benzene-sulfuric acid (20:10:1) [83,84], concentrated, washed with water until being free from acidity, dried over anhydrous sodium sulfate and kept for GC/MS analysis [85].

#### 3.3.3. GC/MS Analysis of Unsaponifiable Matter

The unsaponifiable matter was analyzed via GC/MS, adopting the following instruments and conditions: Shimadzu GC/MS-QP 5050 A; Software Class, 5000; searched library, Wiley 229. LIB; DBI capillary column, 30 m × 0.53 mm i.d. × 1.5 µm film thickness; injector port temperature, 250 °C; detector cell temperature, 300 °C; carrier gas, helium (1 mL/min); ionization mode, 70 eV; temperature programming, isothermal at 70 °C for 2 min, followed by an increase to 220 °C at a rate of 2 °C/min, then remaining isothermal at 220 °C for 5 min.

#### 3.3.4. GC/MS Analysis of Fatty Acid Methyl Esters

The fatty acid methyl esters in the PEE were analyzed using a Hewlett Packard HP-6890 GC-series adopting the following conditions: capillary column, 60 m × 320 μm; stationary phase, HP-Invowax-polyethylene glycol; film thickness, 0.25 μm; injector port temperature, 250 °C; detector cell temperature, 300 °C; carrier gas, nitrogen (30 mL/min); column temperature programming, isothermal at 70 °C for 2 min, followed by an increase to 120 °C with rate 4 °C /min then isothermal for 2 min at 120 °C, followed by increasing the temperature to 240 °C at a rate of 4 °C/min, then remaining isothermal at 240 °C for 13 min.

Identification of hydrocarbons, sterols, triterpenes and fatty acid methyl esters was carried out by comparing their relative retention times with available reference compounds, in addition to comparing mass spectroscopic data on the identified compounds with computerized data and the available literature. Quantization was based on peak area integration and internal normalization methods. The results of this analysis are presented in Table 1 and Table 2.

### 3.4. Preparation of the Methanolic Extract (ME)

A defatted, powdered specimen of *S. bicolor* (50 g) was refluxed with methanol (500 mL) for 2 h. The methanol extract was then filtered and concentrated under reduced pressure to yield a sample of 3 g, which was retained for biological analysis as well as the identification and quantitative estimation of different groups of chemical compounds.

### 3.5. Identification and Quantification of Phenolic Compounds

#### 3.5.1. Determination of the Total Phenol Content

The total phenolic content in the *S. bicolor* methanol extract was determined spectrophotometrically using the Folin-Ciocalteu reagent [29,55]. The reaction mixture contained methanol extract (1 mL, 500 μg/mL), Folin-Ciocalteu reagent (0.5 mL), 20% sodium carbonate (3 mL) and distilled water (10 mL). After 2 h of reaction at ambient temperature, the absorbance at 765 nm was measured and used to calculate the phenolic content, with gallic acid being employed as a standard. The total phenolic contents were expressed as gallic acid equivalents (GAE) in mg/g dry sample. A calibration curve of the standard gallic acid concentration (mg/mL) against the absorbance (nm) was constructed (regression equation, y= 6.026x + 0.039; r^2^ = 0.998). The concentration of the total phenol content in the plant extract was calculated using the expression C= c × (V/m), where C is the total phenolic content of the methanol extract in mg/g (GAE); c is the concentration of gallic acid established from the calibration curve (mg/mL); V is the volume of the extract (mL); and m is the weight of the plant extract (g). The absorbance was measured with a Genesys spectrophotometer (Milton Roy, Inc., Rochester, NY, USA) at 765 nm using methanol as a blank, and the concentration of the total phenolic content of the extract was calculated.

#### 3.5.2. Qualitative and Quantitative HPLC Analysis of Phenolic Acids

HPLC analyses were performed using a Knauer HPLC system (Berlin, Germany) with a model 64-00 pump, model 87-00 UV detector and model 7,125 injection valve (Rheodine, Cotai, CA, USA), and chromatographic separation was performed on a LiChrospher RP-18 (5 mm) column (250 × 4 mm i.d. Merck, Darmstadt, Germany). The solvent system used in these analyses consisted of a gradient of water and acetonitrile at a pH of 2, adjusted with phosphoric acid. The following gradient was used: 0–20 min, water/acetonitrile, 95:5 v/v; 20–40 min, water/acetonitrile, 75:25 v/v; 40–45 min, water/acetonitrile, 1:1 v/v; 45–60 min, water/acetonitrile, 25:75 v/v. The operating conditions were as follows: column temperature, 25 °C; injection volume, 20 µL; flow rate, 1.0 mL/min; and the UV spectra were recorded from 220 to 600 nm.

The following standards were used: *trans*-cinnamic acid, *o*-coumaric acid, *m*-coumaric acid, *p*-coumaric acid, caffeic acid, ferulic acid, syringic acid, synaptic acid, chlorogenic acid, salicylic acid, sorbic acid, sinapic acid, 3-hydroxybenzoic acid, 4-hydroxycinnamic acid, 4-hydroxybenzoic acid, protcatechuic acid, gallic acid, elagic acid and digallic acid, which were obtained from Sigma Aldrich Co. (St. Louis, MO, USA). Each standard phenolic acid sample (2 mg) was dissolved in methanol/water (10 mL, 50:50 v/v), and 20 µL of each standard phenolic acid sample was injected for HPLC analysis under the same conditions. The spectrum of each standard was recorded and stored in the HPLC spectrum library. The criteria for the identification of phenolic compounds were established based on comparison of the retention time and spectrum of an unknown compound with library data on HPLC standards. Quantification was carried out using the external standard method. Solutions of each standard at various concentrations were injected into the HPLC system, and the peak areas were determined. Thus, the calibration curves and response factors were recorded under the same conditions as for the samples. The concentration of a compound was calculated as the peak area of the compound X response factor. The results are presented in Table 3.

#### 3.5.3. Determination of Total Flavonoids

Determination of the total flavonoid content in the *S. bicolor* methanol extract was performed colorimetrically using an aluminum chloride solution [86]. A standard curve was constructed using different concentrations of quercetin in methanol (six serial two-fold dilutions to produce concentrations of 100–3.125 µg /mL). Then, methanol extract (100 µL) was added to a 96-MicroWell plate, followed by 2% aluminum chloride solution in methanol (100 µL). After 10 min, the absorbance was measured with a Genesys spectrophotometer (Milton Roy, Inc., Rochester, NY, USA) at 415 nm using methanol as blank, and the concentration of total flavonoids was calculated.

#### 3.5.4. Qualitative and Quantitative HPLC/MS Analysis of Flavonoid Contents

HPLC analysis of the methanol extract from the aerial parts of *S. bicolor* was performed using a Hewlett-Packard series 1100 system (Waldbronn, Germany) with a symmetry C18 column (250 mm × 4.6 mm, i.d. 4 µm) and a guard column (10 mm × 3.9 mm, i.d. 4 µm) from Waters (Barcelona, Spain), equipped with a vacuum degasser, a binary pump and a photodiode array detector (HP1050), connected to HP ChemStation software (Hewlett-Packard) and an APAL autosampler (CTC analytics) controlled by its own software. Elution was carried out with a gradient of acetonitrile (solvent B) in the form of a 0.05% TFA solution in water (solvent A), and the elution conditions applied were as follows: 0–60 min, linear gradient of 5–50% B; 60–70 min, linear gradient of 50–75% B; 70–80 min, 75–100% B; and 80–90 min, 100% B, isocratic. The flow rate was 0.7 mL/min, and the injection volume was 50 µL. The system was operated at room temperature.

The HPLC was coupled with a Finnigan-MAT model TSQ 700 (San Jose, CA, USA) triple quadrupole mass spectrophotometer equipped with an APCI interface. LC/APCI-MS analyses were performed in positive ion mode. The APCI parameters for the source were as follows: capillary temperature, 200 °C; vaporizer temperature, 450 °C; corona needle current, 5 µA; sheath gas, nitrogen; collision gas, helium; collision energy, 50%. Identification of compounds was accomplished by comparing mass spectroscopic data on the identified compounds with computerized data and the available literature. Quantitative estimation of each flavonoid component was achieved by preparing the ME in triplicate, and each preparation was analyzed in triplicate. The results are presented in Table 4. Standards (apigenin, luteolin, luteolin 7-*O*-glucoside, vitexin, isovitexin, orientin, isoorientin, kaempferol, kaempferol 3-methyl ether, quercetin, salvigenin and quercetin 3,7,3-trimethyl ether) and solvents were purchased from Sigma–Aldrich GmbH (Steinheim, Germany). Calibration curves were constructed for each flavonoid in the range of sample quantities of 0.02–0.5 μg. HPLC grade acetonitrile was obtained from Merck (Darmstadt, Germany). Phosphoric acid (J.T. Baker, Phillipsburg, NY, USA) and redistilled water were used; after preparation of the mobile phases, they were filtered through a 0.49 nm filter. All other chemicals used were of analytical grade.

### 3.6. Evaluation of Antioxidant Activity

#### 3.6.1. DPPH Radical-Scavenging Activity

The hydrogen atom or electron donation ability of the petroleum ether and methanoic extracts from *S. bicolor* was measured based on the bleaching of a purple-colored methanol solution of DPPH [87]. Thus, the antioxidant activity of the PEE and ME was determined on the basis of the scavenging activity of stable 2,2-diphenyl-1-picrylhydrazyl (DPPH) free radicals [55]. For these assays, 0.001 M DPPH in methanol (3 mL) was added separately to the PEE and ME at different concentrations (1 mL, 250, 500, 1,000, 1,500, 2,000 and 2,500 μg/mL). The absorbance at 517 nm was determined after 30 min, and the percent of activity inhibition was calculated as [(A_o_ − A_e_)/A_o_] × 100 (A_o_ = absorbance without extract; A_e_ = absorbance with extract). IC_50_ values were calculated via linear regression of plots, where the abscissa represented the concentration of the tested plant extracts, and the ordinate represented the percent antioxidant activity of the different dilutions of the extracts. 

#### 3.6.2. Determination of Total Antioxidant Capacity

This assay is based on the reduction of Mo (VI) to Mo (V) by the extract and the subsequent formation of a green phosphate/Mo (V) complex at an acid pH [60]. Each extract (0.3 mL) was added to reagent solution (3 mL, 0.6 M sulphuric acid, 28 mM sodium phosphate and 4 mM ammonium molybdate). The tubes were then incubated at 95 °C for 90 min. After the mixture had cooled to room temperature, the absorbance of the solution was measured at 695 nm against a blank. The antioxidant activity was expressed as the number of equivalents of gallic acid (mg GAE/g dry sample), and a calibration curve of the gallic acid concentration (µg/mL) against the absorbance (nm) was established (regression equation, y = 0.092x + 70.49; r^2^ = 0.745).

### 3.7. Biological Analysis

Animals: Sprague-Dawley strain rats or Swiss albino mice of average weight, which was from 100–120 g for rats and 25 g for mice, were used. Food and water were provided *ad libitum*.

#### 3.7.1. Determination of Anti-inflammatory Activity

PEE and ME were evaluated to determine their anti-inflammatory activities using the carrageenan-induced paw edema assay in rats [88]. The animals were divided into four groups, and the effect of oral administration of the PEE and ME from *S. bicolor* aerial parts at doses of 50 mg/kg (0.5 mL, n = 6/group) or indomethacin (10 mg/kg, 0.5 mL) given as a 60 min pretreatment was studied. The control group received saline (0.5 mL, n = 6/group). Paw edema was induced by sub-plantar injection of 100 µL of 1% sterile carrageenan lambdain saline into the right hind paw. The contral ateral paw received an equal volume of saline. Paw volume was determined immediately before carrageenan injection and at selected times thereafter using a plethysmometer (UgoBasile, Milan, Italy). The edema component of inflammation was quantified by measuring the increase in paw volume before carrageenan injection and at 1, 2, 3 and 4 h after carrageenan injection with respect to the pre-injection value for each animal. Edema was expressed as the percentage of change from the control. The results are presented in Table 5.

#### 3.7.2. Determination of Analgesic Activity

The hot plate test was performed on rats using an electronically controlled hot plate (UgoBasile, Italy) heated to 52 °C (± 0.1 °C). The cut-off time was 30 seconds. Groups of rats (n = 6/group) were administered the PEE and ME of *S. bicolor* aerial parts at a dose of 50 mg/kg, saline (control), or indomethacin at 20 mg/kg 30 min prior to testing. The latency to licking a hind paw or jumping out of the apparatus was recorded for the control and drug-treated groups. Pain thresholds were measured sequentially before treatment and at 1 and 2 h post-treatment [89]. The results are presented in Table 6.

### 3.8. Determination of Antimicrobial Activity

Antimicrobial activity was tested using American Type Culture Collection (ATCC) standards against *Staphylococcus aureus* ATCC 13709,*Staphylococcus epidermidis* ATCC 35984, *Streptococcus pyogens* ATCC 10782, *Escherichia coli* ATCC 9637, *Klebsiella pneumonia* ATCC 13883, *Proteus vulgaris* ATCC 6380, *pseudomonas aeruginosa* ATCC 7853,*Shigellaboydii* ATCC 9905, *Candida albicans* ATCC 10231, *Candida glabrata* ATCC 90030, *Candida krusei* ATCC 6258 and *Candida parapsilosis* ATCC 22019. Bacterial strains were cultured overnight at 37 °C in Mueller Hinton agar, and yeasts were cultured overnight at 30 °C in Sabouraud dextrose agar.

The agar disc diffusion method [90] was employed for determination of the antimicrobial activities of the PEE and ME. A suspension of the tested microorganism (0.1 mL of 10^8^ cells per mL) was spread on solid media plates. Aliquots of 15 µg of the PEE and ME dissolved in dimethyl sulfoxide (DMSO, Merck, Germany) were applied on sterile paper discs (6 mm diameter). Gentamycin and amphotericin B were used as standard antibacterial and antifungal agents, respectively, as positive controls, and DMSO without the extracts was used as a negative control. The discs were deposited on the surface of inoculated agar plates. These plates were held at 4 °C for 2 h, followed by incubation at 37 °C for 24 h for bacteria, or at 30 °C for 48 h for yeasts. The diameters of the inhibitory zones were measured in millimeters. All tests were performed in triplicate.

For determination of minimum inhibitory concentrations, a microdilution broth susceptibility assay was used, as recommended by NCCLS [91].Tests for bacterial strains were performed in Mueller Hinton Broth supplemented with Tween 80 detergent at a final concentration of 0.5% (v/v), while those for fungal strains were performed in Sabouraud dextrose broth with Tween 80. The bacterial strains were cultured overnight at 37 °C in Mueller Hinton Broth, and the yeasts were cultured overnight at 30 °C in Sabouraud dextrose broth. Test strains were suspended in Mueller Hinton Broth at a final density of 5 × 10^5^ cfu/mL, which was confirmed by viable counts. Geometric dilutions ranging from 0.035 to 72.0 mg/mL of the PEE and ME were prepared in a 96-well micro titer plate. The plates were incubated under normal atmospheric conditions at 37 °C for 24 h for bacteria, or at 30 °C for 48 h for yeasts. Bacterial growth was indicated by the presence of a white pellet at the well bottom. The results are presented in Table 7.

### 3.9. Statistical Analysis

Results are presented as means ± standard deviation. The statistical analyses of the experimental results were based on the analysis of variance method. Differences were considered significant at the *p* < 0.001 level.

## 4. Conclusions

In conclusion, petroleum ether and methanol extracts from the aerial parts of *S. bicolor* exhibited anti-inflammatory, analgesic, antioxidant and antimicrobial activities. These effects might be attributed to the detected unsaturated fatty acids, sterols and triterpenes in the petroleum ether extract and phenolic acids and flavonoids in the methanol extract. These results showed that *S. bicolor* extracts could be considered as natural antioxidant and antimicrobial agents and to represent a good analgesic and anti-inflammatory remedy.
